# From Breast to Eye: A Rare Case of Ocular Metastasis From Luminal Breast Cancer in a Nicaraguan Patient

**DOI:** 10.7759/cureus.80748

**Published:** 2025-03-17

**Authors:** Gilberto A Altamirano, Christopher Romero, Catherine S Moreno Cabrera, Johanna I Sobalvarro, Lorenzo E Aragón Conrado

**Affiliations:** 1 Gynecologic Oncology, Military Teaching Hospital "Dr. Alejandro Dávila Bolaños", Managua, NIC; 2 School of Medicine, Military Teaching Hospital "Dr. Alejandro Dávila Bolaños", Managua, NIC; 3 Medical Education, Military Teaching Hospital "Dr. Alejandro Dávila Bolaños", Managua, NIC

**Keywords:** breast cancer, choroidal metastasis, ocular metastasis, radiotherapy, supportive and palliative care

## Abstract

Ocular metastases from breast cancer are rare but significant, with invasive lobular carcinoma (ILC) showing a higher tendency for ocular involvement than invasive ductal carcinoma (IDC). Radiotherapy is the main treatment, but the prognosis is poor. This case involves a 37-year-old woman with a history of bilateral breast cancer, initially treated with surgery, chemotherapy, and radiotherapy. She later developed pulmonary progression, bone metastases, and other complications. The patient experienced worsening vision loss, headaches, tinnitus, and systemic decline. Imaging showed brain lesions and choroidal metastasis. Extensive metastasis was confirmed, including in the pleura, liver, and bones. Ophthalmologic evaluation revealed increased intraocular pressure, requiring medical management and planned cyclophotocoagulation. Due to the advanced disease, a palliative care approach was initiated, with radiotherapy planned for ocular metastasis. This case highlights the aggressive nature of metastatic luminal B breast cancer with ocular involvement and underscores the importance of early detection and a multidisciplinary approach to patient care.

## Introduction

Breast cancer remains one of the leading causes of morbidity and mortality among women worldwide. In 2022, approximately 2.3 million new cases and 670,000 deaths were reported globally. While mortality rates have declined in countries with a very high Human Development Index (HDI), the disease burden remains significant in low- and middle-HDI regions, highlighting persistent disparities in access to early detection and treatment [1].

Among metastatic patterns, ocular involvement is a rare but clinically significant manifestation, occurring in approximately 0.5% of breast cancer cases and representing 2.4% of all systemic metastases, with the choroid and orbit being the most commonly affected structures [2,3].

Invasive lobular carcinoma (ILC) exhibits a higher propensity for metastasizing to atypical sites, including the eye, compared to invasive ductal carcinoma (IDC) [4]. In Latin America, breast cancer is the primary malignancy associated with choroidal metastases, surpassing lung cancer [5]. Despite a lower incidence of ILC, women in this region tend to be diagnosed at younger ages than their European counterparts, while hormone receptor-positive metastatic disease remains prevalent across both populations [6].

The standard treatment for ocular metastases from breast cancer is radiotherapy, which can help preserve visual function. However, its presence often signals extensive systemic involvement and a poor prognosis, with high mortality within months of diagnosis [2]. This case report presents a young woman with bilateral breast cancer and progressive metastatic disease, including ocular involvement, emphasizing the critical role of comprehensive diagnostic evaluation and multidisciplinary management in optimizing patient care.

## Case presentation

A 37-year-old female patient with a history of left breast cancer, stage IIB (luminal B, HER2-positive), diagnosed six years ago and treated with breast-conserving surgery, lymph node sampling, chemotherapy, and radiotherapy. One year ago, she was diagnosed with contralateral right breast cancer, stage IA, managed with a modified radical mastectomy.

Her medical history is also significant for a retinal detachment in the left eye, which was treated with cyclophotocoagulation in March 2024. She currently exhibits pulmonary disease progression due to lymphangitic carcinomatosis and is undergoing chemotherapy with trastuzumab/emtansina for pulmonary involvement and zoledronic acid for bone metastases. She has a documented prednisone allergy and is receiving oncologic pain management, including morphine 20 mg/mL (5 mg subcutaneously every six hours), gabapentin 300 mg orally at bedtime, and prednisone 5 mg orally once daily.

The patient presented to her oncology unit for the scheduled 12th cycle of chemotherapy; however, treatment was deferred due to the onset of new neurological and systemic symptoms over the previous week. She reported progressive blurred vision leading to complete vision loss in the left eye, severe oppressive frontoparietal headaches (9/10 intensity), tinnitus, nausea, generalized malaise, and fatigue. Additionally, 10 days prior, she noted submaxillary lymphadenopathy and exertional dyspnea significantly limiting her daily activities. On physical examination, her BMI was 24.55, and she had experienced an unintentional weight loss of 13.6 kg over the past 7.5 months. Vital signs were within normal limits. However, a firm, mobile submaxillary lymph node enlargement of 2 cm in diameter was detected. Pulmonary examination revealed bilateral absence of vesicular breath sounds at the lung bases, with dullness on percussion, consistent with pleural involvement

Ophthalmologic evaluation of the right eye showed a cup-to-disc ratio of 0.4 mm, a round and well-defined optic disc, an attached retina with diminished macular reflex, and inferior lattice degeneration at the 8 o'clock position, with a visual acuity of 20/50. In contrast, the left eye exhibited no light perception, a cup-to-disc ratio of 0.4 mm, a round optic disc with diminished macular reflex, and an inferonasal retinal detachment without posterior pole involvement.

**Figure 1:**
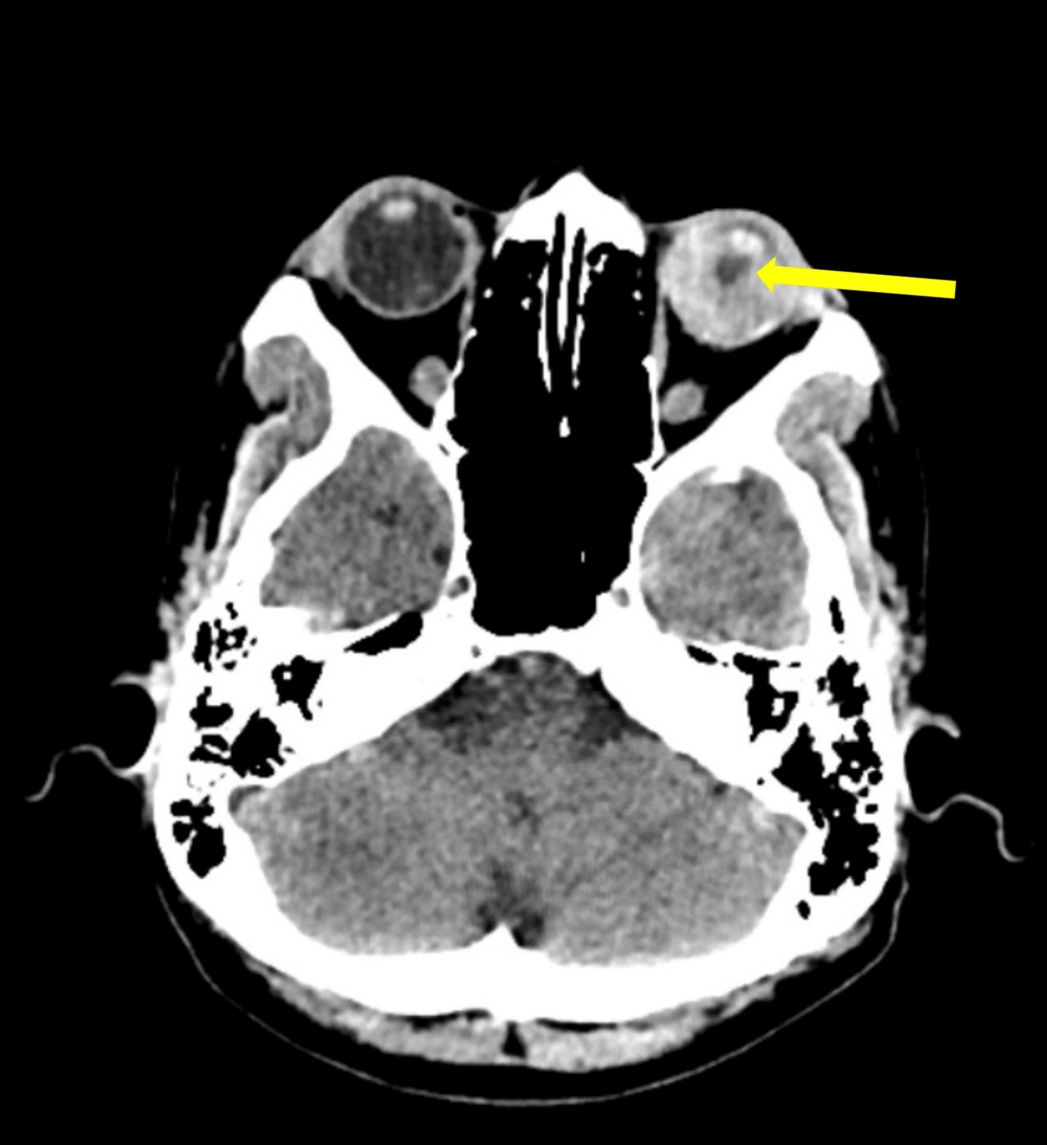
. Cranial computed tomography. Yellow arrow indicating hemorrhage in the left eye's posterior chamber.

The patient's clinical presentation raised concerns for a potential oncologic complication, prompting hospital admission. Given the complexity of her case and prior medical history, an extensive diagnostic evaluation was undertaken, including paraclinical testing (Table 1) and cranial computed tomography. Imaging revealed nodular lesions within the left parietal lobe and thalamus, exhibiting radiological features highly suggestive of metastatic disease. Additionally, a probable choroidal metastasis was identified, accompanied by hemorrhage within the left posterior chamber (Figure 1). These findings strongly correlate with the patient’s oncologic history and reinforce the suspicion of disease progression.

**Table 1:** Laboratory tests

Laboratory Test	Results	Reference Range
Hematocrit	37.1%	39-50%
Hemoglobin	13 g/dL	13-17 g/dL
White blood cell count	5.14 × 10³/µL	4-11 × 10³/µL
Platelet count	232 × 10³/µL	150-500 × 10³/µL
Neutrophil percentage	79.8%	55-65%
Lymphocyte percentage	10.3%	25-35%
Creatine kinase (CK)	87 U/L	30-200 U/L
Creatinine	0.62 mg/dL	0.7-1.2 mg/dL
Potassium	3.8 mmol/L	3.5-5.1 mmol/L
Sodium	135.7 mmol/L	136-145 mmol/L
Aspartate aminotransferase	28.93 U/L	0-39 U/L
Alanine aminotransferase	17.53 U/L	7-63 U/L

Chest computed tomography (CT) revealed significant disease progression, characterized by a subcutaneous tumor implant in the anterior right thoracic wall, increased blastic bone lesions, bilateral pleural effusion, a subpleural nodule in the left upper lobe, and interlobular septal thickening indicative of carcinomatous lymphangitis. Abdominal imaging confirmed metastatic blastic lesions in the spine and pelvis, along with a nodule in hepatic segment VIII exhibiting features consistent with metastasis. These findings highlight the extensive multi-organ involvement and the severity of the patient's current clinical condition.

An ultrasound was performed to quantify the pleural effusion, identifying 200 mL of fluid, which did not meet surgical criteria. Due to the elevated risk associated with thoracentesis, loop diuretic therapy was initiated, as potassium levels and renal function were within acceptable parameters for its safe administration.

A consultation with the ophthalmology service was requested to rule out choroidal metastasis. During the evaluation, intraocular pressure was measured using a Goldmann tonometer, recording 12 mmHg in the right eye and 29 mmHg in the left eye. To reduce intraocular pressure, treatment was initiated in the left eye with dorzolamide 2%, brimonidine 0.2%, and timolol 0.5% (one drop of each every 12 hours). A consultation with a glaucoma sub-specialist was indicated, who recommended a cyclodestructive procedure using a transscleral cyclophotocoagulation laser, achieving intraocular pressure control at 13 mmHg in the left eye.

**Figure 2:**
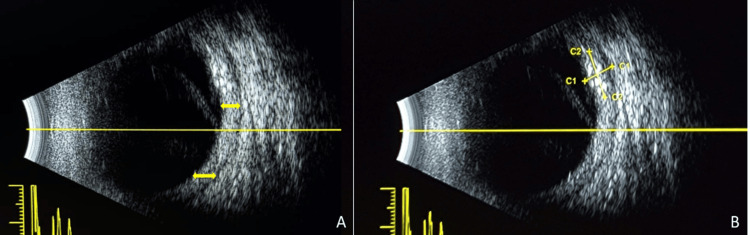
Left eye ultrasound. A. Yellow arrows indicate choroidal thickening. B. Yellow arrows show the dimensions of the mass: 3.27 mm × 4.95 mm.

A retinography was also attempted; however, visualization of ocular structures was hindered by abundant exudative material. Optical coherence tomography (OCT) was subsequently performed but failed to produce satisfactory imaging, prompting the use of ocular ultrasonography. Real-time ultrasonographic imaging in A- and B-modes focused on the posterior chamber of the left eye. The findings demonstrated mobile, diffuse echoes consistent with the presence of erythrocytes or exudative material in the vitreous chamber, as well as a thickened, retracted, echogenic line indicative of an inferior bullous retinal detachment with associated choroidal thickening. Furthermore, a 3.2 mm × 4.9 mm echogenic protuberance was identified in the superior quadrant, suggestive of a metastatic implant, consistent with the patient’s underlying primary disease (Figure 2A-2B).

Given the progression of the disease, a multidisciplinary palliative care plan was developed in collaboration with clinical oncology, pain management, and palliative care services. Morphine doses were adjusted to optimize pain control. Follow-up care was coordinated with both the oncology and ophthalmology services, with a follow-up appointment scheduled in two months to coincide with the completion of the ongoing chemotherapy cycles. Radiotherapy was planned as part of the treatment strategy, along with additional cranial and thoracoabdominal computed tomography scans to evaluate disease progression.

## Discussion

As a subtype of invasive breast cancer (IBC), luminal breast cancer is characterized by the expression of hormone receptors, particularly estrogen receptor (ER) and progesterone receptor (PR), and is typically HER2-negative. The luminal subtype is divided into two distinct categories: luminal A and luminal B, each with its own clinical and pathological features [7-8].

Luminal A tumors are typically ER-positive, PR-positive or negative, HER2-negative, and exhibit low proliferation rates (Ki-67 index), which correlates with a more favorable prognosis. On the other hand, luminal B tumors, although also ER-positive, often require more intensive treatment, owing to their higher proliferation rates, potential HER2 positivity, and increased axillary lymph node involvement [7-10].

The most common histological subtype of luminal breast cancer is invasive ductal carcinoma (IDC), followed by invasive lobular carcinoma (ILC). Less common subtypes include mucinous carcinoma, tubular carcinoma, and invasive micropapillary carcinoma [11, 12].

Ocular metastasis from breast cancer is an uncommon manifestation, occurring in approximately 0.5% of patients with breast cancer and accounting for 2.4% of all metastatic cases. This condition shows a stronger association with ILC compared to IDC [2]. Our case also aligns with the literature, showing evidence of ocular involvement in a patient with a history of breast cancer; however, it was not identified as invasive lobular carcinoma, as presented in the literature. Instead, the patient had a more common histology subtype, which further highlights the variability in clinical presentations and the importance of considering ocular metastases in all breast cancer patients, regardless of histological subtype.

Tumor dissemination to the eye can affect multiple ocular structures, including the orbit, choroid, optic nerve, and iris. A notable proportion of cases involve bilateral ocular metastases [1, 10]. The mean age of patients diagnosed with ocular metastases is reported to be approximately 50 years. Importantly, ocular metastasis may represent the first clinical indication of metastatic breast cancer in some patients, highlighting the need for heightened awareness of visual disturbances as potential harbingers of systemic disease [2, 13]. This clinical aspect is highly relevant in our case, where the patient presented with ocular symptoms that were initially attributed to other causes. This delayed recognition of ocular metastasis in our case mirrors findings in previous literature, emphasizing the need for vigilance when dealing with such clinical manifestations.

The common clinical manifestations of ocular metastasis include reduced visual acuity, blurred vision, and ocular pain. In certain cases, ocular metastases are incidentally detected during magnetic resonance imaging (MRI) [2, 14, 15]. The prognosis for patients with ocular metastases is generally poor, with a reported median survival of 10 months after diagnosis [2, 15]. However, other studies have documented a median survival of 32.4 months following the diagnosis of ocular metastasis, highlighting variability in clinical outcomes [3]. Our case, although presenting with ocular metastasis, showed a slightly more favorable prognosis than expected, as the patient responded to the initial therapeutic approach better than the median survival rates mentioned in the literature.

The diagnostic approach to ocular metastases typically involves a combination of clinical examination and imaging studies. MRI and computed tomography (CT) are instrumental in identifying orbital soft tissue masses. Nevertheless, a definitive diagnosis requires histopathological confirmation. The primary therapeutic objective is to preserve visual acuity and enhance the patient's quality of life, despite the generally unfavorable prognosis associated with the condition [16, 17]. In our case, MRI was pivotal in identifying ocular involvement, which led to appropriate management. The treatment approach in our case included radiation therapy, similar to what has been suggested in the literature, though the patient’s response was better than typically expected for ocular metastasis.

Radiotherapy is the cornerstone treatment for ocular metastases; however, systemic therapies and multimodal approaches are also commonly employed [2, 3]. Despite these efforts, the therapeutic response remains heterogeneous, with some patients achieving only partial symptomatic relief. In cases involving concurrent metastases to other organs, systemic treatments such as chemotherapy and hormonal therapies are integral components of care [16, 17]. These complexities underscore the necessity of a multidisciplinary approach to managing this patient population [13]. In our case, the multidisciplinary team was critical, as the patient’s response to hormonal therapy was better than expected, potentially contributing to an improved overall outcome.

Addressing the unique challenges of ocular metastases in luminal breast cancer is pivotal for optimizing patient outcomes and advancing therapeutic strategies. Ocular metastases in luminal breast cancer pose unique challenges due to extended latency periods and the limitations of current prognostic tools. Traditional mammography has drawbacks, particularly in women with dense breast tissue, highlighting the need for more effective, non-invasive diagnostic methods. Liquid biopsy, analyzing biomarkers in body fluids such as blood, urine, sweat, and breath, has gained attention for early cancer detection. Advances in "omics" research have identified circulating tumor markers, microRNAs, and extracellular vesicles as promising diagnostic tools. Future developments in liquid biopsy could revolutionize early detection and monitoring of breast cancer, improving patient outcomes [18]. Our case demonstrates the potential for new diagnostic methods like liquid biopsy, though it was not part of the initial diagnostic workup. This suggests that integrating newer diagnostic tools could help improve early detection, especially in cases of ocular metastasis where traditional imaging may miss early ocular involvement.

Molecular classification through gene expression profiling, such as PAM50, is instrumental in distinguishing luminal subtypes, predicting clinical outcomes, and guiding treatment decisions. This classification underpins the shift toward personalized therapeutic strategies. Additionally, emerging evidence on miRNA expression profiles highlights their potential to refine diagnostic, prognostic, and therapeutic approaches in luminal A breast cancer. These findings underscore the critical need for further research into the integration of precision medicine into routine clinical practice [12, 19]. Our case aligns with the notion of personalized therapy but also highlights the gaps in current research regarding ocular metastasis in luminal breast cancer. As our understanding of molecular subtypes advances, integrating these insights into clinical practice could help tailor therapies more effectively.

## Conclusions

Secondary ocular metastasis from breast cancer, while infrequent, represents a significant clinical challenge due to its profound functional implications and its association with a poor prognosis. This case report highlights the necessity of a thorough and systematic evaluation in patients with a history of malignancy and atypical visual symptoms. Notably, invasive lobular carcinoma demonstrates a higher predisposition for metastasis to uncommon sites, including the eye. The presented clinical findings and imaging studies illustrate the inherent complexity of this condition and underscore the indispensable role of advanced diagnostic modalities in achieving early and accurate detection of metastatic disease.

A multidisciplinary approach encompassing radiotherapy, systemic chemotherapy, and palliative care remains the cornerstone of treatment aimed at improving the quality of life and mitigating symptoms in these patients. Nevertheless, the rapid disease progression observed in such cases highlights an urgent need for the development of more effective and individualized therapeutic interventions. This case contributes valuable insights to the growing body of literature on the metastatic patterns of breast cancer within the Latin American context, further emphasizing the global importance of understanding regional variations. Future research should prioritize the identification of prognostic biomarkers and the implementation of non-invasive diagnostic techniques to enhance clinical management and optimize patient outcomes in this population.

## References

[1] Kim J, Harper A, McCormack V (2025). Global patterns and trends in breast cancer incidence and mortality across 185 countries. Nat Med.

[2] Yousef YA, Mohammad M, Khalil H (2024). Ocular and periocular metastasis in breast cancer: clinical characteristics, prognostic factors and treatment outcome. Cancers (Basel).

[3] Chang C, Shang Y, Gao Y, Shang M, Wang L, Li H (2022). Clinical features, treatment, and prognosis of 16 breast cancer patients with ocular metastases. Cell Mol Biol (Noisy-le-grand).

[4] Blohmer M, Zhu L, Atkinson JM (2020). Patient treatment and outcome after breast cancer orbital and periorbital metastases: a comprehensive case series including analysis of lobular versus ductal tumor histology. Breast Cancer Res.

[5] Salcedo-Villanueva G, Medina-Andrade AA, Moreno-Paramo D (2021). Primary cancer sites and clinical features of choroidal metastasis in Mexican patients. Clin Ophthalmol.

[6] Kadys A, Gremke N, Schnetter L, Kostev K, Kalder M (2023). Intercontinental comparison of women with breast cancer treated by oncologists in Europe, Asia, and Latin America: a retrospective study of 99,571 patients. J Cancer Res Clin Oncol.

[7] Erber R, Hartmann A (2020). Histology of luminal breast cancer. Breast Care (Basel).

[8] de Lima JB, Silva I, de Oliveira JR (2024). Molecular subtypes of breast cancer: clinicopathological characteristics and prognosis. Concilium.

[9] Johnson KS, Conant EF, Soo MS (2021). Molecular subtypes of breast cancer: a review for breast radiologists. J Breast Imaging.

[10] Pellegrino B, Hlavata Z, Migali C (2021). Luminal breast cancer: risk of recurrence and tumor-associated immune suppression. Mol Diagn Ther.

[11] Volos L, Dudash A (2021). Clinical and morphological features of luminal A subtype of invasive ductal breast cancer. Scientific Journal of Polonia University.

[12] Yamsun M, Imaniah I, Maulana SS, Setiawan RH (2024). Age and luminal type play important role as therapeutic policies in the management of breast cancer at Prof. Dr. Margono Soekarjo Regional Hospital Purwokerto. International Journal of Medical Science and Clinical Research Studies.

[13] King b, Legenza M, Krutzler D (2024). Ocular metastases in breast cancer: a multidisciplinary approach to diagnosis and management. Marshall J Med.

[14] Tsui M, Liu H (2022). Anterior chamber metastases from breast cancer. BMJ.

[15] Tamura M, Tada T, Tsuji H (2004). Clinical study on the metastasis to the eyes from breast cancer. Breast Cancer.

[16] Belhouari M, Khalfi S, Bourhafour M (2022). Ocular metastasis from mammary carcinoma: a case report. Int J Med Rev Case Rep.

[17] Sassi S, Hassouni F, Benbella L (2023). Ocular metastasis from breast carcinoma: a case report. SAS J Med.

[18] Li J, Guan X, Fan Z (2020). Non-invasive biomarkers for early detection of breast cancer. Cancers (Basel).

[19] Kudela E, Samec M, Koklesova L (2020). miRNA expression profiles in luminal A breast cancer-implications in biology, prognosis, and prediction of response to hormonal treatment. Int J Mol Sci.

